# Methods to Explore Changes in the Extent of Habitat Provided by *Ceratophyllum demersum* Shoots for Epiphytic Organisms in Changing Environments

**DOI:** 10.1002/ece3.71612

**Published:** 2025-06-30

**Authors:** Kitti Németh, Attila I. Engloner

**Affiliations:** ^1^ HUN‐REN Centre for Ecological Research Budapest Hungary; ^2^ National Laboratory for Water Science and Water Security HUN‐REN Centre for Ecological Research Budapest Hungary

**Keywords:** aquatic ecology, biofilm, phytomacrofauna, phytoremediation, plant surface, submerged macrophytes

## Abstract

In aquatic environments, the surface of submerged plants provides extensive habitat for a variety of epiphytic organisms; however, appropriate methods to determine this quantity and its changes related to altering environmental conditions are lacking. In this study, we examined one of the most morphologically complex, worldwide distributed submerged macrophytes, *Ceratophyllum demersum* . After exploring the morphological diversity of this plant in various aquatic habitats and accurately measuring its total surface area, we proposed methods to easily calculate or estimate this trait, either based on variables that can be recorded in the field without damaging the plants or on data obtained from the literature. The total plant surface area of the examined shoots with an average total length of 73–143 cm and 28–63 internodes was 147–313 cm^2^; the largest entire plant included in this study had a total surface area of 3352 cm^2^. The greatest morphological diversity in *C. demersum* was caused by variability in the number and total length of internodes, while the internodal diameter and the leaf whorl area varied less. The former two variables are easy to determine; the latter two can provide constants for calculations. In addition, strong correlations were revealed between the total surface area and shoot length, as well as fresh weight, allowing easy estimation of plant surface area and its changes in response to a changing environment. As an example, a change in shoot length of 10 cm or fresh weight of 100 g results in a change in total shoot surface of approximately 15 or 5400 cm^2^. In addition to studies focusing on epiphytic communities, the proposed methods can provide fundamental information for many scientific and practical fields from ecology to phytoremediation and wastewater treatment.

## Graphical abstract

Macrophytes, no information has yet been published on changes in their surface, that is, the extent of their habitat provided for epiphytic organisms. The aim of this paper was to (i) test the morphological diversity of *C. demersum* stands from various aquatic habitats, (ii) develop methods for accurate measurement, calculation, and simple estimation of shoot surface areas, either based on field‐recorded variables or data published in the literature, and (iii) explore how plant surface area can change in changing habitats. The proposed methods can provide fundamental information for many scientific and practical fields, from ecology to phytoremediation and wastewater treatment.

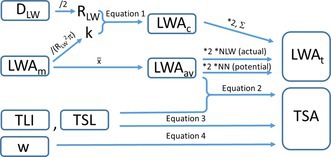

**Abbreviations**
D_LW_
: largest leaf whorl diameter
D_LWav_
: the average diameter of leaf whorls
D_S_
: stem (internodal) diameter
LI: length of the internodes
LI_av_
: the average length of internodes
LLS_av_
: the average length of lateral shoots
LWA: leaf whorl area
LWA_av_
: average LWA calculated from all LWA_m_ data
LWA_c_
: LWA calculated from the radius of leaf whorl
LWA_m_
: LWA digitally measured in microscope image
LWA_t_
: total area of the leaf whorls
NI: number of internodes (NN‐1)
NILS_av_
: the average number of internodes of lateral shoots
NLS: the total number of lateral shoots
NLW: number of leaf whorls
NN: number of nodes
R_LW_
: radius of leaf whorl
TLI: Total length of internodes
TSA: total surface area of shoots
TSL: Total stem length
w: fresh shoot weight

## 1 Introduction

Submerged macrophytes play diverse roles in aquatic ecosystems, many of which are closely related to their structural and morphological characteristics. Stands of these plants can reduce water velocities, promote sedimentation and retention of fine materials, and thereby increase water transparency (Pluntke and Kozerski 2003; Greenway 2007; Hilt 2015; Drexler et al. 2021). Due to the complex architecture and resulting altered environmental conditions (Cotton et al. 2006), macrophytes are diverse and complex habitats for freshwater organisms (Pelicice et al. 2008; Chen et al. 2011; Ferreiro et al. 2013; Wolters et al. 2018; Baattrup‐Pedersen et al. 2025). Many functions performed by macrophytes in aquatic habitats are related to their surface. Through the latter, they can accumulate significant amounts of various elements and hazardous substances directly from the water, thereby removing them from water bodies (Keskinkan et al. 2004; Polechońska et al. 2018; Chen et al. 2019; Xu et al. 2019; Hak et al. 2020; Azeez 2021; Qadri et al. 2022; Wang et al. 2023). Submerged plant parts are natural substrates for epiphytic organisms, ranging from algae and microorganisms (which can form a thick biofilm layer and greatly enhance the removal of element from the water) to various taxa of phytomacrofauna (Pettit et al. 2016; Fan et al. 2021; Engloner et al. 2022; Geng et al. 2022; Deng et al. 2024).

The morphological characteristics that determine the ecosystem functions listed above are highly dependent on environmental conditions and are vulnerable to habitat degradation. For example, it is well known that changes in water depth, often a result of hydrological changes caused by global climate change and other anthropogenic interventions (Alizadeh‐Choobari et al. 2016; Mahdian et al. 2023), affect the shoot length of aquatic macrophytes (Zhu et al. 2012, 2018; Wang et al. 2016; Li et al. 2020; Chen et al. 2024). However, no information has yet been published on how changing environments affect the surface of submerged macrophytes (i.e., the extent of the habitat provided for epiphytic organisms). One reason for this may be that methods for determining the surface area of plant organs or even entire shoots involve many difficulties.

The weighted image technique calculates the surface area from the weight of the copy paper made of plant leaves (Gregg and Rose 1982; Brown and Manny 1985; Sher‐Kaul et al. 1995). During the wetted layer method and colorimetric method, the plant parts are immersed in a liquid or a dye solution and the weight of the thin layer of the surface film covering it is measured, or in the latter method, the color of the water is measured with a spectrophotometer after the dye has been washed from the surface of the plant into a known volume of water (Harrod and Hall 1962; Armstrong et al. 2003; Glenn et al. 2012). These two methods are time‐consuming and overestimate the surface area of plants with segmented leafed plants due to capillary retention of liquid in crevices (Bergey and Getty 2006). Furthermore, the viscosity of the solution depends on the temperature, and its adhesion depends on the quality of the plant surface.

The planimeter is also a tool for determining the size of mostly simple, plain leaves (Spence et al. 1973; Brown et al. 1988; Armstrong et al. 2003), but delineating segmented leaves takes a long time or is not feasible. This technique has been replaced by digital leaf area meter and measurement with image processing software after scanning (Brown and Manny 1985; Hansen et al. 2011; Pettit et al. 2016; Hao et al. 2023), which is fast, but less applicable to species with branched, overlapping, segmented leaves. Furthermore, it gives inaccurate results when the geometry of the different plant parts is not taken into account and both the laminar and cylindrical organs (i.e., leaves and stems) are multiplied by two to obtain the total surface area (Brown and Manny 1985; Brown et al. 1988; Ferreiro et al. 2013).

Simplifying organs into geometric shapes (cylinders or cylinders with conical tips) and mathematically calculating their surface area can also be a solution, but only for a limited number of species, such as macrophytes belonging to the genera *Myriophyllum* and *Ranunculus* (Harrod and Hall 1962; Sher‐Kaul et al. 1995; Warfe et al. 2008). In the case of water stargrass ( *Heteranthera dubia* ), the leaves were considered as isosceles triangles with the bases attached to each other (Balci and Kennedy 2003). The success of this method clearly depends on how similar the chosen geometric shape is to the original shape of organs. The usefulness of any published surface measurements is further reduced if the data are limited to leaves or a part of the shoot (e.g., the top) (Ferreiro et al. 2013; Levi et al. 2015; Pettit et al. 2016), and if the intraspecific morphological diversity is ignored.

Unfortunately, there is an inverse relationship between the morphological complexity of macrophytes and the measurability of their surface. Leaf area of large, simple leaves is easier to measure than many small and/or compound leaves, especially if the latter overlap a lot. However, it is the more morphologically complex plants that have a greater impact on aquatic ecosystems through the above detailed processes (Hinojosa‐Garro et al. 2010; Hansen et al. 2011; do Nascimento Filho et al. 2021).

One of the most morphologically complex macrophytes is *Ceratophyllum demersum* , a cosmopolitan submerged species with wide climatic tolerance. Its richly branched shoots and dichotomously forked leaves arranging in whorls form large plant masses close to the water surface or the entire water column in shallow waters. This species provides all of the ecosystem functions listed above related to morphological complexity, and its surface hosts biofilm‐forming microorganisms and many other epiphytic organisms (Cattaneo et al. 1998; Engloner et al. 2023; Li et al. 2024). So far, only a few studies have dealt with determining the surface area of this species, some of which were hampered by the methodological difficulties detailed above or focused only on plant parts, but not on the whole shoots (Brown et al. 1988; Armstrong et al. 2003; Ferreiro et al. 2013; Pettit et al. 2016). The lack of a simple and suitable method for determining the surface area of *C. demersum* also means that we still lack information on how the changes in environmental characteristics may affect this crucial feature for epiphytic organisms.

The aim of this study was to (i) test the morphological diversity of *C. demersum* stands from various aquatic habitats, (ii) develop methods for accurate measurement, calculation and simple estimation of shoot surface areas, and (iii) explore how surface area can change in habitats where environmental characteristics change.

## 2 Material and Methods

### 2.1 Study Sites

**FIGURE 1:**
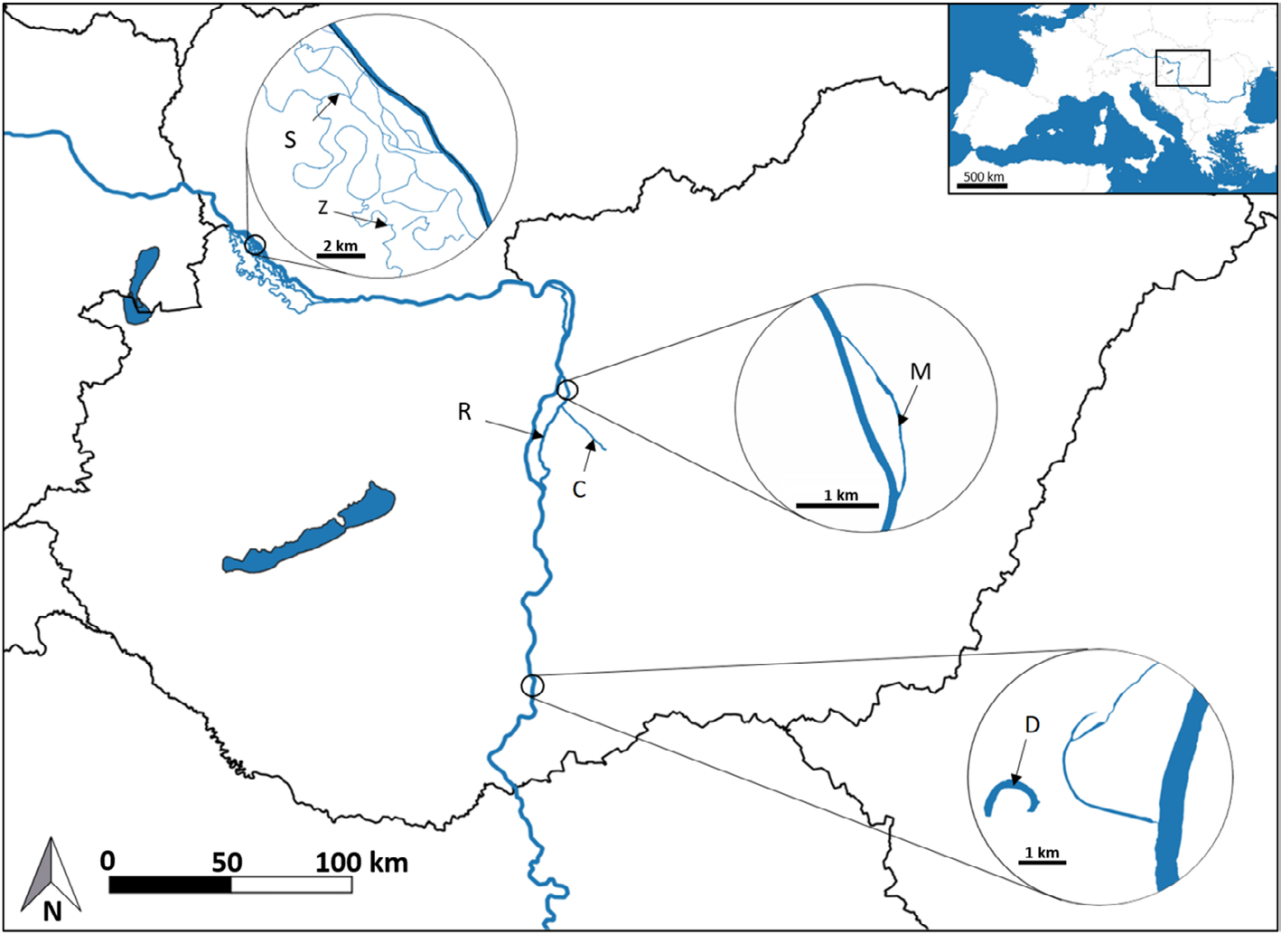
Sampling locations. C, Danube‐Tisa Channel; D, Decsi‐Nagy Danube; M, Molnár Island branch; R, Ráckeve‐Soroksár Danube; S, Schisler oxbow; Z, Zátonyi Danube.

Plant samples were collected from six different aquatic habitats (Figure 1). Ráckeve‐Soroksár Danube (R) is the second largest side arm of River Danube in Hungary between the 1642 and 1586 river kms. Its water regime is regulated by sluices at both ends. The 2 km‐long Molnár Island branch (M) is connected to the northern section of Ráckeve‐Soroksár Danube and is characterized by slow water flow. The island and the tributary are both Natura 2000 areas in Budapest. The Danube‐Tisza Channel (C) is a 22 km long canal that originates from the Ráckeve‐Soroksár Danube and is primarily used for irrigation and drainage of internal water. It is characterized by low or absent water flow. Schisler oxbow (S) and Zátonyi Danube (Z) are located in the Szigetköz, an important wetland area with many branches, islands, and backwaters, at the interfluve of the main Danube channel and the Moson arm. Schisler oxbow is in the active floodplain, while Zátonyi Danube is located in the flood‐protected area. Decsi‐Nagy Danube (D) is a former Danube side branch lying on the floodplain of Gemenc Landscape Protection Area, which can be temporarily connected to the Danube during the river's floods. The water quality parameters of the six habitats are presented in Table 1.

**TABLE 1:** Water quality parameters of the six habitats (mean ± SD).

	R	S	M	D	Z	C
WD (cm)	133.2 ± 26.9	153.1 ± 46.7	62.1 ± 3.2	37.9 ± 5.8	204.1 ± 60.6	101.3 ± 10.1
T (°C)	24.3 ± 0.2	25.4 ± 0.6	23.0 ± 0.9	24.4 ± 3.9	25.5 ± 2.7	23.8 ± 0.7
pH	7.9 ± 0.1	7.6 ± 0.2	7.7 ± 0.5	9.1 ± 0.5	7.6 ± 0.4	7.6 ± 0.1
EC (μS/cm)	382.5 ± 11.8	351.50 ± 8.4	512.1 ± 82.3	299.2 ± 30.1	299.3 ± 19.4	454.7 ± 2.1
DO (mg/L)	11.1 ± 0.8	7.05 ± 1.4	4.5 ± 3.1	13.7 ± 2.8	5.5 ± 0.5	5.7 ± 1.8
Redox (mV)	183.5 ± 4.7	181.0 ± 4.6	162.6 ± 42.3	213.8 ± 4.1	158.2 ± 34.7	185.7 ± 4.2

*Note:* R, S, M, D, Z and C are habitat abbreviations as given in Figure 1.

Abbreviations: DO, dissolved oxygen; EC, electric conductivity; Redox, redox potential; T, water temperature; WD, water depth.

### 2.2 Sampling and Measurements

For morphological comparison and surface measurements, shoots anchored into the sediment were collected, keeping a distance of at least 30 m between two samples, so the number of plants (replicates) taken from each habitat was determined by the size of the water bodies and the amount of plant cover in them. All collected plants were rinsed with water from the sampling points, placed into plastic bags, and then transported wet and cool. After a thorough examination in the laboratory, incomplete plants, that is, those that were missing shoot tips, were excluded. Plants with damaged or missing leaf whorls along the shoots were retained, but both the actual (i.e., existing) and potential leaf whorl numbers were taken into account (see later). A total of 58 shoots were involved in the measurements.

For the morphological evaluations, the number of internodes, the length of the internodes and the largest diameters of each leaf whorl were determined on the main shoots. The number of internodes is always one less than the number of nodes, as the uppermost node only carries a measurable leaf whorl, and the internode below the lowermost node is incomplete and therefore does not provide usable length data. From the diameters of leaf whorls, their radii were also calculated and used in the following. In addition, the number and length of lateral shoots of the main shoots, as well as the number of the internodes of these lateral shoots were also determined. The length of the internodes was measured without the nodal region, that is, between the leaf bases of successive leaf whorls.

**FIGURE 2:**
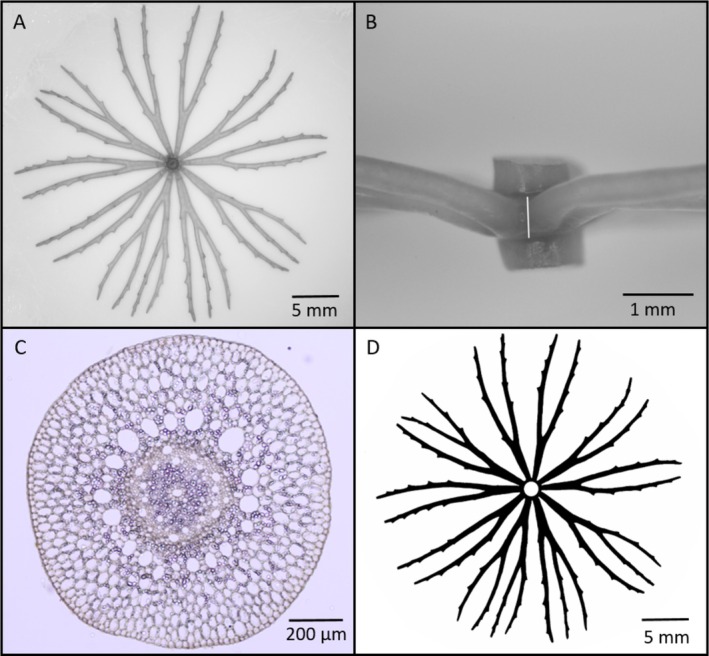
Images used for morphological and anatomical measurements. (A) Adaxial surface of a leaf whorl; (B) Side view of a leaf whorl with a white line indicating the axial length of node; (C) Cross‐section of an internode; (D) Binary image of the adaxial surface of a leaf whorl without node cross‐sectional area.

After that, the main stems were cut with a razor into leaf whorls (with associated nodes) and internodes. Under a stereo microscope (Olympus SZX7), all remaining debris and filamentous algae were removed from the plant parts with tweezers and a dissecting needle. Using a Canon EOS 1200D digital camera attached to the microscope, each leaf whorl was digitized from two directions: from above (Figure 2A) and from the side (Figure 2B). In the former images, the area of leaf whorls (hereinafter denoted as LWA_m_) and the cross‐sectional area of the node were measured, while in the latter, the axial thickness of the nodal region was determined. Since the nodes are covered by the bases of the leaves, the surface of this region belongs to the surface of leaf whorls. If the total stem length (TSL, i.e., internodes and nodes together) is used for calculations (see later), the surface area of the nodes is considered twice, resulting in an overestimation of the total plant surface. Determining the thickness and then the surface area of nodal regions allows the calculation of the degree of this overestimation.

To determine stem diameter (D_s_), the middle part of every fifth internode of the shoots was embedded into glycol methacrylate resin (Leica Historesin, Heidelberg, Germany), from which 4 μm thin cross‐sections were made with a microtome (Leica RM2265). Cross sections were also digitalized with the same camera attached to a light microscope Olympus BX43 (Figure 2C). Stem diameter (five per sections), node area and thickness, and leaf whorl area (the latter after converting the images into binary, Figure 2D) were measured using ImageJ software. The leaf whorl areas were determined without the cross‐sectional areas of the nodes. During the evaluation of the data, only the measurements of intact leaf whorls were taken into account, a total of 75.

### 2.3 Data Evaluation and Surface Area Calculation and Estimation

To compare the morphology of *C. demersum* plants from the six habitats, the following variables were calculated for each main shoot and evaluated with both multivariate and univariate tests: the average length of internodes; the average diameter of leaf whorls; the total number of lateral shoots; the average length of lateral shoots; and the average number of internodes of lateral shoots. Canonical variates analysis (CVA) was performed with the SYN‐TAX 2000 computer program package (Podani 2001). To test significant differences between variables, Tukey's HSD (Honestly Significant Difference) tests were carried out. Differences with *p* values under 0.05 were considered significant.

To detect vertical differences in shoot morphology across the six habitats, the length of the uppermost 20 internodes and the largest diameters of the uppermost 20 leaf whorls of the main shoots were also compared using Tukey's HSD.

**FIGURE 3:**
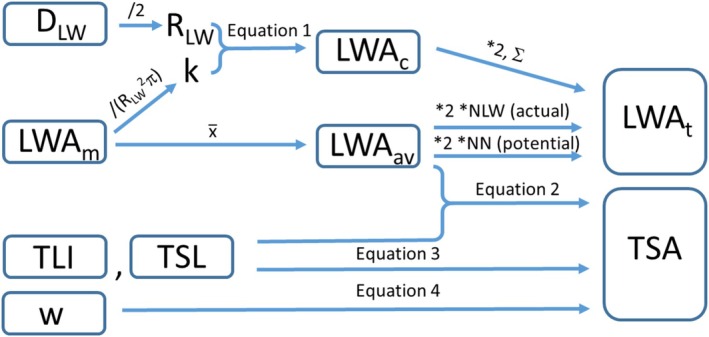
Schematic summary of the main surface area calculations and estimations.

To determine the surface area easily and quickly, we performed the following calculations and estimates (for ease of understanding, a schematic summary of these is shown in Figure 3). For each leaf whorl, we determined the ratio of their digitally measured area (LWA_m_) to the area of the circle calculated from the radius of the leaf whorl. The average of this ratio was used as the constant *k* to calculate the area of the leaf whorl (LWA_c_) from their radius, as follows:

(1) $$\mathrm{LW}A_{c}={R_{\mathrm{LW}}}^{2}\times \pi \times k$$

where, *R*_LW_, radius of leaf whorl; *k*, constant (i.e., the average ratio of individual leaf whorl areas and the circle areas with R_LW_). Based on all the measurements (all LWA_m_ data), we also determined an average leaf whorl area (LWA_av_).

The total area of the leaf whorls (LWA_t_) in a shoot was determined as

- The twice the sum of leaf whorl areas (LWA_c_) calculated individually according to Equation (1), and to make the calculation quicker and easier,
- The twice of average leaf whorl area (LWA_av_) multiplied by the number of leaf whorls (NLW).

The relationships between the diameter and measured area of leaf whorls (DLW and LWA_m_), the measured and calculated leaf whorl areas (LWA_m_ and LWA_c_), as well as the total leaf whorl areas (LWA_t_) determined from individual calculations (LWA_c_) and the average leaf whorl area (LWA_av_) were examined with Pearson's correlation tests and linear regressions.

When the number of leaf whorls was used for the calculations, both the actual number of existing leaf whorls (NLW) and their total potential number (equal to the number of nodes, NN) were taken into account. The former can be used to determine the actual total surface area, while the latter is suitable for specifying the total potential surface (i.e., as if all the leaf whorls were intact, that is, none were damaged or missing). Based on these two calculations, we determined how much surface area loss was caused by leaf whorl damage occurring in the habitats.

Finally, the total surface area (TSA) was calculated as follows:

(2) $$\mathrm{TSA}=L\times D_{s}\times \pi +N\times 2\times \mathrm{LW}A_{\mathrm{av}}$$

where, L, the total length of internodes (i.e., the sum of internode length measurements, TLI) or the total stem length, TSL, including internodes and nodes; D_s_, diameter of stem; N, number of leaf whorls (NLW); LWA_av_, average leaf whorl area.

This equation was applied to the main shoots and their 20 cm long sections from top to bottom. For each section, we also calculated the ratio of its surface area to the total area. Also using Equation (2), the surface area of the entire plants (i.e., the main shoot together with the lateral shoots) was also determined. In this case, L and N represented the total length of the branch system and the total number of its leaf whorls (nodes).

Finally, Pearson's correlation tests and linear regressions were performed between the total surface area and the shoot length and weight. The results of these tests provided an additional possibility (Equations 3 and 4, see later) to roughly but simply estimate the shoot surface area of *C. demersum* .

### 2.4 Estimation of Shoot Surface Areas Based on Literature Data

To demonstrate differences between shoot surface areas developing at different water depths, we applied Equation (3) (derived from the relationship between total shoot length and surface area, see Results section) to data from studies that revealed significant differences in shoots height depending on water depth. Wang et al. (2016) examined the growth of *C. demersum* in controlled plot experiments and recorded significantly different shoots in water depth of 30, 90 and 150 cm. Zhu et al. (2018) studied this species along three water depth gradients in a lake habitat and also found significant differences in shoot lengths at different water depths, however, shoots developing at the same water depths in different transects were not necessarily of the same length. To determine the TSA of plant mass per m^2^ of habitat area, we applied Equation (4) (i.e., the relationship between the fresh weight and surface area of shoots, see Results section) to the data presented by Nikolić et al. (2007).

## 3 Results

### 3.1 Morphological Diversity of *C. demersum* Stands

**FIGURE 4:**
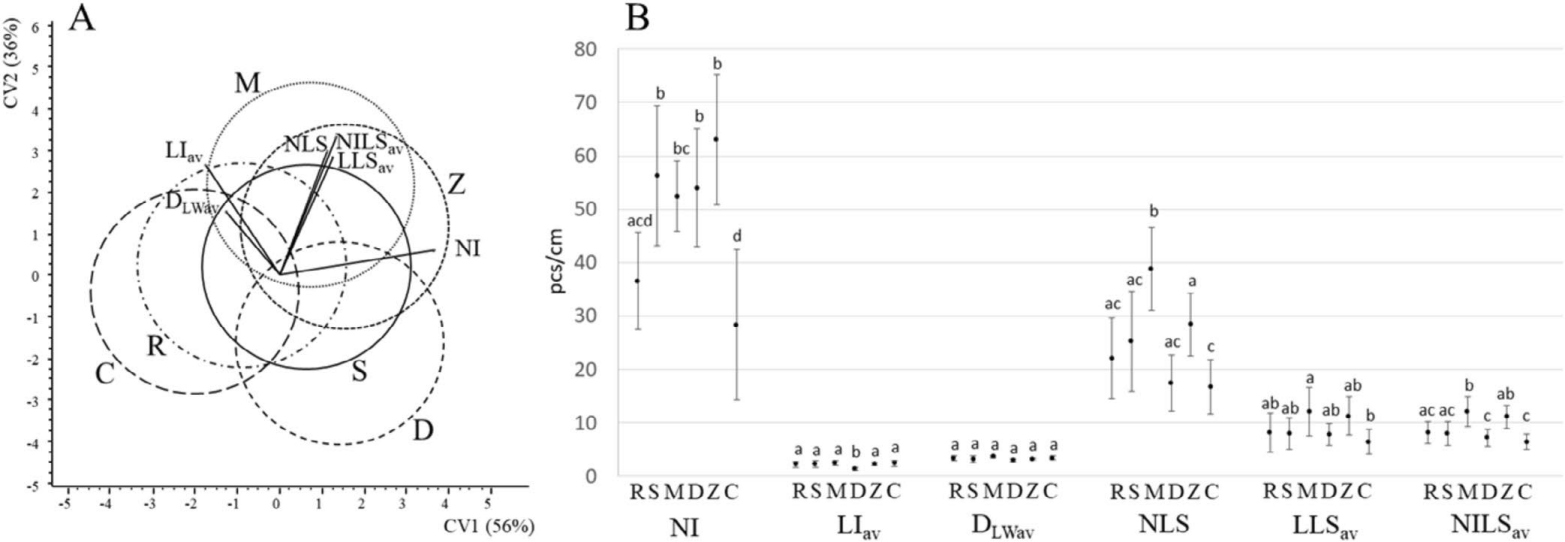
Morphological differences of *C. demersum* shoots from the six habitats. (A) CVA biplots with 95% isodensity circles; (B) Mean value of variables (± SD) per habitats with significant differences based on Tukey tests (*p* < 0.05). R, S, M, D, Z and C are habitat abbreviations as given in Figure 1.

Based on their morphology, multivariate statistical analysis showed large overlaps between the shoots of *C. demersum* from the six habitats (Figure 4A). The strongest separation (along the first canonical variable, CV1) was caused by the number of internodes, represented by a long vector most parallel to CV1. Further separations (along CV2) were mainly due to morphological characteristics of the lateral shoots. The significance tests led to the same result; the number of internodes and the morphology of the lateral shoots differed the most between the habitats (Figure 4B). These variables also had high within‐habitat variance. In contrast, the average diameter of leaf whorls and the mean length of internodes did not differ or hardly differed between the habitats. On average, main shoots consisted of 37, 56, 52, 54, 63, and 28 internodes, with an average length of 2.25, 2.27, 2.48, 1.39, 2.27, and 2.40 cm, resulting in a total length of 83, 126, 130, 76, 143, and 73 cm in habitats R, S, M, D, Z, and C, respectively. The average diameter of leaf whorls was 3.32, 3.18, 3.62, 3.00, 3.20, and 3.42 cm.

The comparison of the internode lengths in the top 20 positions of the shoots (Figure A1) showed that this variable can vary strongly within and between habitats. However, this variability was mostly observed in the lower positions, and the length data of the uppermost 1–6 internodes were more similar. The largest diameter of the leaf whorls of the six habitats did not differ significantly in the majority of vertical positions (Figure A1).

Internodal diameters precisely determined on cross‐sections showed little variation within and between habitats (Table 2). The mean value of this variable calculated from all measurements of all habitats was 1.317 mm, which was later used as D_s_ in Equation (2). Although leaf whorl area (LWA_m_) was more variable than internode diameter, it did not differ significantly among the six habitats either (Table 2). The mean measured area of leaf whorls for all measurements in all habitats (i.e., LWA_av_ used in Equation 2) was 198.32 mm^2^.

**TABLE 2:** Differences in internodal diameters and surface areas from the six habitats (mean ± SD).

	R	S	M	D	Z	C
D_S_ (μm)	1222.9 ± 235.9 a	1242.9 ± 233.4 a	1331.2 ± 254.9 a	1367.0 ± 235.5 a	1460.9 ± 325.1 a	1213.6 ± 117.9 a
LWA_m_ (mm^2^)	178.9 ± 132.5 a	276.3 ± 33.9 a	183.5 ± 82.8 a	231.4 ± 91.1 a	196.3 ± 66.8 a	153.9 ± 59.7 a
Total LWA form LWAav (cm^2^)	136.5 ± 41.2 ac	188.9 ± 50.0 b	176.1 ± 33.0 ab	203.7 ± 48.1 b	209.4 ± 40.6 b	101.8 ± 33.0 c
TSA of the main shoots (cm^2^)	183.0 ± 45.9 ad	279.3 ± 64.2 c	265.5 ± 34.5 abc	249.6 ± 52.8 abc	313.1 ± 59.1 c	146.7 ± 80.1 d
TSA of plants (cm^2^)	941.7 ± 432.5 ac	1014.5 ± 475.4 ac	1947.5 ± 850.2 b	733.7 ± 277.7 a	1531.7 ± 424.9 bc	557.7 ± 220.2 a

*Note:* R, S, M, D, Z and C are habitat abbreviations as given in Figure 1. Different letters indicate significant differences (*p* < 0.05).

### 3.2 Correlations Between Measured and Calculated Variables

**FIGURE 5:**
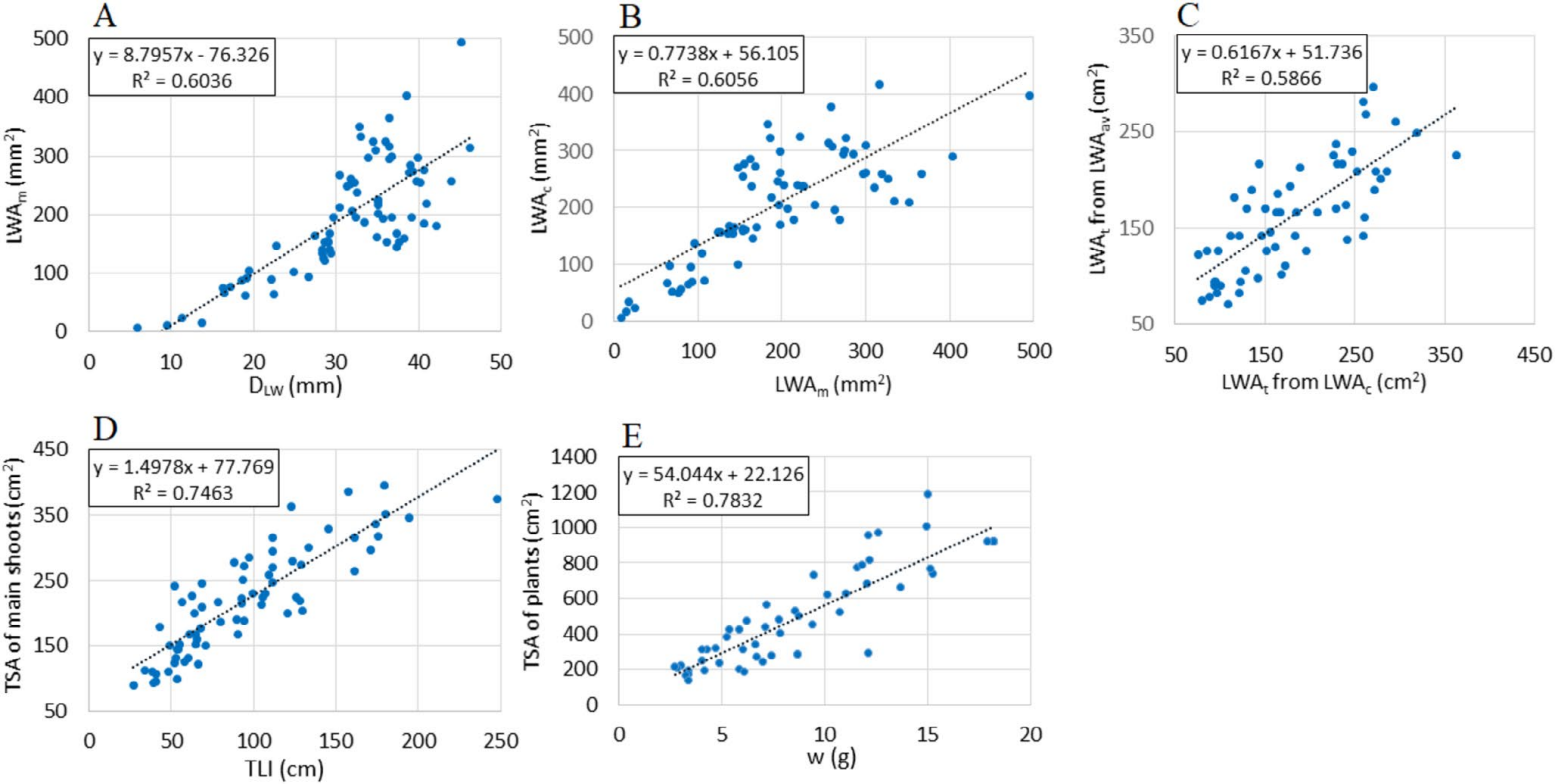
Linear regressions of measured or calculated characteristics. Detailed statistics of regressions are given in Table A1.

The correlation between the diameter and the measured area of leaf whorl (D_LW_ and LWA_m_) was positive and strong (with a coefficient of 0.777); the result of the linear regression is presented in Figure 5A. (The statistics for this and subsequent regressions are provided in Table A1.) The average ratio of LWA_m_ to the circle area calculated from the radius of the leaf whorl was 0.250 (SD = 0.072). Therefore, this value was used as the constant *k* in Equation (1) to calculate leaf whorl area from its radius. LWA_c_ showed a positive and strong correlation with LWA_m_ (the coefficient was 0.778). Figure 5B shows the result of the linear regression.

When determining the total surface area of the leaf whorls of the main shoots (LWA_t_), a strong positive correlation (with a coefficient of 0.765) was revealed between the sum of the individually calculated leaf whorl area values (LWA_c_) and the total area calculated based on the average leaf whorl area (LWA_av_). The result of the linear regression is shown in Figure 5C.

The TSA of the main shoots showed a strong positive correlation with the total internodal length; the coefficient was 0.846, the result of linear regression is presented in Figure 5D. So, TSA (in cm2) can be roughly estimated from the total stem length, using Equation (3):

(3) TSA=1.5×TSL+77.8

where TSL is the total stem length in cm.

This relationship also allows us to estimate the extent to which the surface area changes with the changes in the shoot length. As Equation (3) suggests, the change in shoot length (in cm) multiplied by 1.5 gives the change of surface area in cm^2^.

A strong correlation was also observed between the TSA and fresh weight of the shoots (with a coefficient of 0.885). The result of the linear regression is presented in Figure 5E. According to this, TSA (in cm^2^) can also be roughly estimated from the fresh weight of the shoots, using Equation (4):

(4) TSA=54×w+22.1

where *w* is the fresh weight of the shoots in g.

This equation also helps to estimate the extent to which the surface area changes with the changes in the fresh weight of the shoot. The change in the latter (in g) multiplied by 54 gives the change of surface area in cm^2^.

### 3.3 The Total Surface Areas of Leaf Whorls, Shoots, Shoot Segments, and Whole Plants

The mean total surface area of leaf whorls was 136.5, 188.9, 176.1, 203.7, 209.4, and 101.8 cm^2^ in habitats R, S, M, D, Z, and C, respectively (Table 2). Calculation for the total potential surface area (i.e., as if all the leaf whorls were intact, that is, none were damaged or missing) gave 7%–21% higher values: 148.7, 227.0, 211.8, 218.2, 253.8, and 116.3 cm^2^ for the same habitats.

The total surface area (TSA) of the main shoots varied between 146.7 and 313.1 cm^2^ on average, showing a large within‐habitat variation and no significant differences between most habitats (Table 2). The pattern of differences between habitats follows that shown by the number of internodes (cf. Figure 4B).

The degree of overestimation of the TSA when including the stem length in Equation (2) instead of the sum of internodal lengths (i.e., when considering the area of the nodes twice) was about 0.5%, since the axial thickness of nodes were around 615 μm.

Comparison of the TSA of 20 cm long shoot sections showed that, regardless of total length, *C. demersum* shoots had the largest surface area at the top (in our case, the uppermost 20 cm long sections gave 70–114 cm^2^) and the values decreased downwards (Table 3). Of course, the longer the shoot, the smaller the proportion of the upper surfaces compared to the total surface area.

**TABLE 3:** Average surface area (cm^2^) of shoot sections and its percentage (in brackets) compared to the total shoot surface.

Total length of shoots (cm)	Average TSA of shoots (cm^2^)	20 cm long shoot sections from top to bottom											
		Top 20	20–40	40–60	60–80	80–100	100–120	120–140	140–160	160–180	200–220	220–240	240 <
≤ 40	106	70 (66)											
40 ≤ 60	145	76 (53)	41 (29)										
60 ≤ 80	186	93 (49)	46 (25)	35 (19)									
80 ≤ 100	227	89 (39)	44 (19)	36 (16)	37 (17)								
100 ≤ 120	267	114 (43)	53 (19)	31 (12)	25 (10)	26 (10)							
120 ≤ 140	259	93 (35)	35 (13)	32 (12)	26 (10)	26 (10)	33 (13)						
140 ≤ 160	358	109 (30)	42 (11)	36 (10)	30 (8)	28 (8)	36 (10)	48 (14)					
160 ≤ 180	309	99 (32)	39 (13)	34 (11)	28 (9)	22 (7)	21 (7)	26 (8)	25 (8)				
180 ≤ 200	364	97 (26)	31 (8)	28 (8)	24 (7)	19 (5)	25 (7)	23 (6)	32 (9)	73 (20)			
240 ≤	376	80 (21)	20 (5)	28 (7)	24 (6)	20 (5)	20 (5)	20 (5)	28 (7)	28 (7)	24 (6)	36 (10)	36 (10)

*Note:* The shoots are grouped along their total length (rows). The higher the percentage, the darker the color.

The TSA of the plants (i.e., the combined TSA of the main shoots and all their lateral shoots) averaged between 558 and 1948 cm^2^ (Table 2), with the minimum and maximum of 246 and 3352 cm^2^. The largest surface areas were found in habitats M and Z, exactly where the most lateral shoots were recorded (cf. Figure 4B).

### 3.4 Estimated Shoot Surface Areas Based on Literature Data

TSA estimates of *C. demersum* shoots based on literature data are given in Table 4. The longest shoot was recorded by Zhu et al. (2018), with a length of 349 cm, providing a surface area of approximately 602 cm^2^. It can be seen that if the length of a shoot increases from 100 to 200 cm (or decreases from 200 to 100 cm) due to altering water depth, its total surface area changes from 228 to 378 cm^2^ (or vice versa). As shown in the data in Table 4, the differences between surface areas (in cm^2^) are equal to the shoot length differences (in cm) multiplied by 1.5.

**TABLE 4:** TSA estimates of *C. demersum* shoots based on literature data.

	Water depth (cm)	Shoot length (cm)	Shoot fresh weight per m^2^ (g)	TSA (cm^2^)
Wang et al. (2016)	30	100 a		228
	90	200 b		378
	150	300 c		528
Zhu et al. (2018)	50	93 a		217
	150	135 bc		280
	250	272 f		486
	350	272 f		486
	450	349 g		602
	150	147 bcd		299
	250	192 e		366
	350	292 f		515
	50	103 a		232
	150	131 b		275
	250	176 de		342
	350	167 cde		328
	450	192 e		366
Nikolić et al. (2007)			1880	101,542
			2200	118,822
			1755	94,792
			1320	71,302
			1050	56,722
			324	17,518
			537	29,020
			448	24,214

*Note:* Different letters indicate significant differences between shoot length data tested by the cited authors.

According to Nikolić et al. (2007), *C. demersum* shoots can reach 324–2200 g fresh weight per m^2^ of habitat, which means total surface areas of 17,518–118,822 cm^2^. The differences between surface areas (in cm^2^) are equal to the shoot fresh weight differences (in g) multiplied by 54.

## 4 Discussion

In this study, we investigated one of the most morphologically complex submerged species with richly branched shoots and highly segmented leaves in whorls (Best and Visser 1987; Ahmad et al. 2016; Les 2017; Xu and Deng 2017). First, we made accurate measurements and tested the morphological variability within and between populations. The morphological comparison of six different *C. demersum* populations showed that internodes differed the most between the habitats, while the diameter of leaf whorls changed less and did not show statistically significant differences between habitats and mostly vertically in shoots. High variability in internode and total shoot length has been previously shown for several submerged macrophytes (Aiken 1981; Santamaría et al. 2003; Jiang et al. 2008; Xie et al. 2013) and specifically also for *C. demersum* (Hyldgaard et al. 2012; Liu et al. 2018).

The strong positive correlation revealed between the diameter and the digitally measured area of leaf whorls allows the calculation of the area from the radius of the leaf whorls using a constant. In this way, the area of a leaf whorl is 0.25 times the area of the circle calculated from the radius of the leaf whorl. A further simplification is to calculate with the average value of the surface area of leaf whorls. The strong positive correlations between the digitally measured and calculated leaf whorl areas, and the total leaf whorl surfaces of shoots determined by the sum of individual calculations and using the mean leaf whorl area value support the goodness of the surface estimates obtained by the simplified calculations.

Due to its cylindrical shape, it seems easy to determine the surface area of the stem from the length and the diameter, but some difficulties may arise during measurements. On the one hand, unless we have information on the invariance of the internode diameter vertically in a shoot and between shoots, a very large number of individually performed measurements are required. On the other hand, if the total stem length (as a single measurement from bottom to top) is used for the calculations, the surface of nodes is considered twice, because the nodes are covered by leaf bases and the surface of this region is included in the surface of the leaf whorl. Stem diameter invariance has been demonstrated in some submerged macrophytes (Chatenet et al. 2006; Jiang et al. 2008; Riis et al. 2010), and we also found small diameter variability in the internodes of *C. demersum* within and between habitats. These results support the use of the mean stem diameter value. We also showed that if the entire stem length is included in the surface calculation instead of the individual internode measurements, the overestimation is small, around 0.5%.

After the above, the TSA can be easily and very quickly calculated based on four data points: the total shoot length, the mean internode diameter (1.317 mm), the number of leaf whorls, and the average leaf whorl area (LWA_av_, 198.32 mm^2^). Since many leaf whorls are located near the apex (Les 2017; Gargiulo et al. 2022), this is where a large part of the surface area of *C. demersum* is located. According to our results, the lengths of internodes are also the most similar in this part of the shoots.

Due to the strong correlations between shoot length and fresh weight, and total surface area, the latter can be quickly estimated from the former two. This also means that surface changes resulting from changes in shoot length or weight can also be determined. *C. demersum* most often occurs in free‐floating form in water (Best and Visser 1987), the plant mass can be returned to its habitat without damage after weighing or determining the total shoot length.

Of course, all the simplifications presented result in a certain degree of inaccuracy, as do all estimates. However, the use of average values calculated from non‐ or less variable morphological characteristics and the high correlation between the measured and calculated variables provide good estimates that can be obtained quickly even in the field, without damaging the plants, and therefore may be more suitable for many studies than the precise but time‐consuming measurements of all plant parts.

The provided equations can also be applied to data published in the literature, so further post‐experimental information can easily be obtained based on them. The morphology of submerged macrophytes is influenced by many environmental characteristics in addition to water depth, such as light availability, turbidity, and temperature (Barko et al. 1982; Riis et al. 2012; Zhu et al. 2018; Yang et al. 2022; Chen et al. 2024), so in most cases it is difficult or impossible to find a single background variable behind morphological changes. By examining plants from different environments, our study showed that the morphological variability of *C. demersum* is manifested mainly in changes in the number of nodes (and also leaf whorls and internodes) and the lengths of internodes, and hardly in stem diameter and leaf whorl morphology. So our method helps to estimate the extent of plant surface and its changes in a diverse and changing environment. As an example, a 10 cm change in shoot length or a 100 g change in fresh weight results in a change of approximately 15 or 5400 cm^2^ in total shoot surface.

Determining the plant surface area and its changes can be crucial not only for studies focusing on epiphytic organisms, but also for physiological experiments expressing CO_2_ assimilation on an area basis (Righetti et al. 2007), or for estimating the accumulation of elements through the plant surface in phytoremediation and metal‐contaminated wastewater treatment (Rai et al. 1995; Lesage et al. 2007; Polechońska et al. 2018; Hak et al. 2020; Ge et al. 2023).

Although the calculations presented in this study are applicable only to a single, albeit globally distributed species, *C. demersum* , the methods of constructing the equations may be suitable for other submerged macrophytes.

## 5 Conclusions

Although many functions of macrophytes in aquatic ecosystems are closely related to structural and morphological characteristics, quantifying these features can involve many difficulties, from the method used to the intraspecific morphological diversity. In addition, the more compound the leaves, the more difficult it is to quantify, for example, its surface area.

Considering the morphological variability of *C. demersum* , its total plant surface area can be easily determined, in addition to precise measurement, either based on variables recorded even in the field or estimated based on shoot lengths or mass data. The proposed methods are also suitable for estimating the extent of plant surface changes caused by altered environmental characteristics and provide fundamental information for many scientific and practical fields, from ecology to phytoremediation and wastewater treatment.

## Author Contributions

**Kitti Németh:** data curation (supporting), formal analysis (supporting), investigation (lead), methodology (supporting), visualization (equal), writing – original draft (equal). **Attila I. Engloner:** conceptualization (lead), data curation (lead), formal analysis (lead), funding acquisition (lead), investigation (supporting), methodology (lead), resources (lead), visualization (equal), writing – original draft (equal).

## Conflicts of Interest

The authors declare no conflicts of interest.

## Supporting information


**Appendix S1.** Supporting Information.

**Appendix S2.** Supporting Information.

## Data Availability

Reviewer URL: https://doi.org/10.5061/dryad.573n5tbmc.
