# Supplementary material for: Methods to Explore Changes in the Extent of Habitat Provided by Ceratophyllum demersum Shoots for Epiphytic Organisms in Changing Environments
Source: Ecol Evol. 2025 Jun 30;15(7):e71612. doi: 10.1002/ece3.71612 (PMC12207483; doi:10.1002/ece3.71612)
Supplement: Supplementary file 1 — Appendix S1. Supporting Information. [file ECE3-15-e71612-s002.docx]

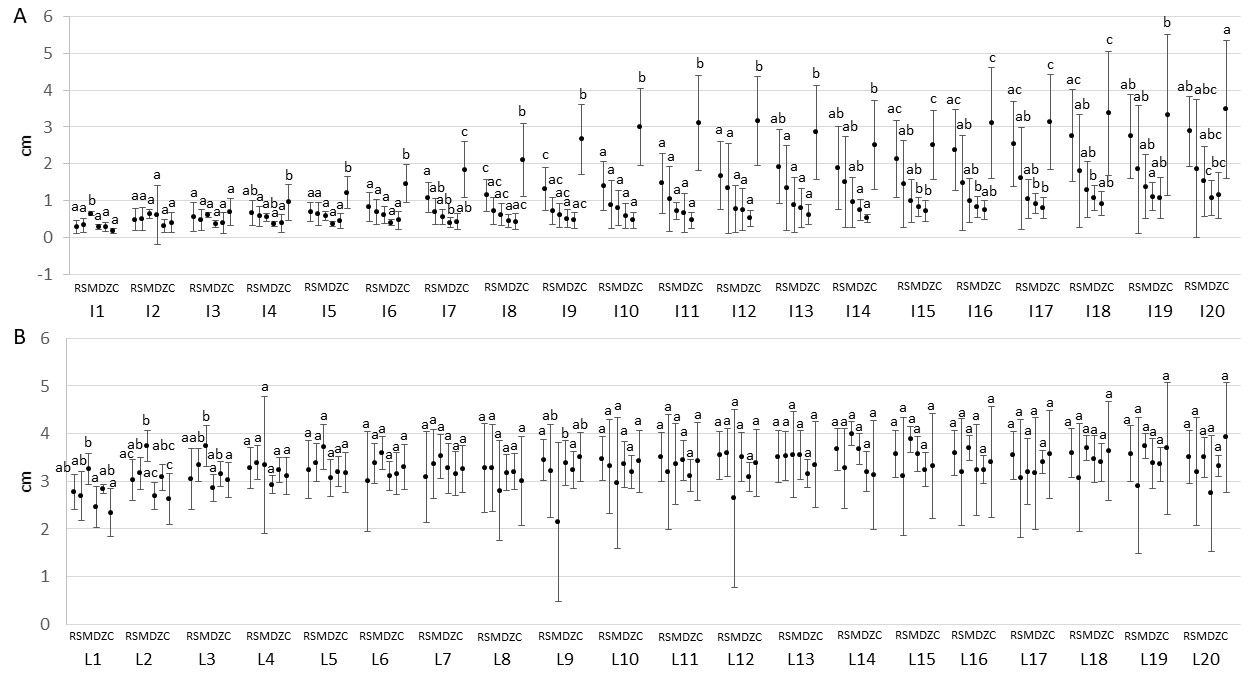


FigA1. Differences in the upper 20 internode lengths (A) and leaf whorl diameters (B) of the shoots from the six habitats. I – internode length, L – leaf whorl diameter, position 1 is the top. R, S, M, D, Z and C are habitat abbreviations as given in Fig.1.
