# Supplementary material for: Methods to Explore Changes in the Extent of Habitat Provided by Ceratophyllum demersum Shoots for Epiphytic Organisms in Changing Environments
Source: Ecol Evol. 2025 Jun 30;15(7):e71612. doi: 10.1002/ece3.71612 (PMC12207483; doi:10.1002/ece3.71612)
Supplement: Supplementary file 2 — Appendix S2. Supporting Information. [file ECE3-15-e71612-s001.docx]

Table A1. Statistics of the regressions shown in Fig.5.

|  | Regressions | | | | |
| --- | --- | --- | --- | --- | --- |
|  | A | B | C | D | E |
| R Square | 0.6036 | 0.6056 | 0.5866 | 0.7463 | 0.7832 |
| Standard Error | 61.3467 | 61.3291 | 37.1289 | 40.6478 | 132.4672 |
| Significance F | 2.54411E-16 | 2.24499E-15 | 2.48211E-12 | 1.2471E-21 | 6.7572E-19 |
| Coefficients-Intercept | -76.326 | 56.105 | 51.736 | 77.769 | 22.126 |
| Standard Error-Intercept | 26.995 | 16.333 | 13.453 | 11.211 | 39.471 |
| t Stat-Intercept | -2.827 | 3.435 | 3.846 | 6.937 | 0.561 |
| P-value-Intercept | 0.0061 | 0.0010 | 0.0003 | 1.9661E-09 | 0.578 |
| Lower 95%-Intercept | -130.127 | 23.513 | 24.785 | 55.391 | -57.078 |
| Upper 95%-Intercept | -22.526 | 88.698 | 78.686 | 100.147 | 101.329 |
| Coefficients-X variable | 8.796 | 0.774 | 0.617 | 1.498 | 54.044 |
| Standard Error-X variable | 0.834 | 0.076 | 0.069 | 0.107 | 3.943 |
| t Stat-X variable | 10.544 | 10.218 | 8.914 | 14.037 | 13.706 |
| P-variable-X variable | 2.54411E-16 | 2.24499E-15 | 2.48211E-12 | 1.2471E-21 | 6.7572E-19 |
| Lower 95%-X variable | 7.133 | 0.623 | 0.478 | 1.285 | 46.132 |
| Upper 95%-X variable | 10.458 | 0.925 | 0.755 | 1.711 | 61.956 |

The letters denoting regressions are the same as in Fig.5.
